# Neonatal Resveratrol and Nicotinamide Riboside Supplementations Sex-Dependently Affect Beige Transcriptional Programming of Preadipocytes in Mouse Adipose Tissue

**DOI:** 10.3389/fphys.2019.00083

**Published:** 2019-02-08

**Authors:** Madhu Asnani-Kishnani, Ana M. Rodríguez, Alba Serrano, Andreu Palou, M. Luisa Bonet, Joan Ribot

**Affiliations:** ^1^Grup de Recerca Nutrigenòmica i Obesitat, Laboratori de Biologia Molecular, Nutrició i Biotecnologia, Universitat de les Illes Balears, Palma de Mallorca, Spain; ^2^CIBER de Fisiopatología de la Obesidad y Nutrición, Palma de Mallorca, Spain; ^3^Institut d’Investigació Sanitària Illes Balears, Palma de Mallorca, Spain

**Keywords:** adipose tissue, beigeing, browning, metabolic programming, sex differences, food bioactives, primary culture

## Abstract

Nutritional programming of the thermogenic and fuel oxidation capacity of white adipose tissue (WAT) through dietary interventions in early life is a potential strategy to enhance future metabolic health. We previously showed that mild neonatal supplementations with the polyphenol resveratrol (RSV) and the vitamin B3 form nicotinamide riboside (NR) have sex-dependent, long-term effects on the thermogenic/oxidative phenotype of WAT of mice in adulthood, enhancing this phenotype selectively in male animals. Here, we tested the hypothesis that these dietary interventions may impact the commitment of progenitor cells resident in the developing WAT toward brown-like (beige) adipogenesis. NMRI mice received orally from postnatal day 2–20 (P2–20) a mild dose of RSV or NR, in independent experiments; control littermates received the vehicle. Sex-separated primary cultures were established at P35 from the stromovascular fraction of inguinal WAT (iWAT) and of brown adipose tissue (BAT). Expression of genes related to thermogenesis and oxidative metabolism was assessed in the differentiated cultures, and *in vivo* in the iWAT depot of young (P35) animals. Neonatal RSV and NR treatments had little impact on the animals’ growth during early postnatal life and the expression of thermogenesis- and oxidative metabolism-related genes in the iWAT depot of young mice. However, the expression of brown/beige adipocyte marker genes was upregulated in the iWAT primary cultures from RSV supplemented and NR supplemented male mice, and downregulated in those from supplemented female mice, as compared to cultures derived from sex-matched control littermates. RSV supplementation had similar sex-dependent effects on the expression of thermogenesis-related genes in the BAT primary cultures. A link between the sex-dependent short-term effects of neonatal RSV and NR supplementations on primary iWAT preadipocyte differentiation observed herein and their previously reported sex-dependent long-term effects on the thermogenic/oxidative capacity of adult iWAT is suggested. The results provide proof-of-concept that the fate of preadipocytes resident in WAT of young animals toward the beige adipogenesis transcriptional program can be modulated by specific food bioactives/micronutrients received in early postnatal life.

## Introduction

Brown adipocytes in canonical BAT depots in mammals express uncoupling protein 1 (UCP1) and a high oxidative capacity and mitochondria content, which distinguishes them metabolically from white adipocytes in typical WAT depots. A third type of adipocytes, beige adipocytes (also called brite [brown-in-white]), are brown-like adipocytes found in WAT depots. Beige adipocytes share with brown adipocytes a common core thermogenesis gene signature including inducible UCP1 expression, which allows for the regulated dissipation as heat of the energy contained in fatty acid and glucose fuel molecules (Bartelt and Heeren, 2014). Additionally, transcripts of several markers have been identified that appear to be specific for beige adipocytes (Wu et al., 2012). The emergence of beige adipocytes in WAT is termed WAT browning or beigeing. BAT activation and WAT browning are both viewed as potential therapeutic targets in the management of obesity and related metabolic disorders such as hyperlipidemia and diabetes (Bonet et al., 2013; Harms and Seale, 2013).

The recruitment of brown and beige adipocytes responds to a variety of hormonal and diet-related stimulus including the intake of specific food bioactives (Bonet et al., 2013, 2017). This is well established in adult rodents and appears to hold for humans as well (Rossato et al., 2014; Fleckenstein-Elsen et al., 2016; Palmeri et al., 2016; Saito et al., 2016). Besides contemporary dietary intake in adulthood, exposure to food bioactives in prenatal and early postnatal life might be important. Epidemiological and experimental evidence indicates that early life nutrition can influence metabolic health in adulthood through programming effects on the developing tissues, with consequences in the long-term (Cottrell and Ozanne, 2008). The browning potential of WAT might be nutritionally programmed (Palou et al., 2013), as well as other aspects such as WAT cellularity (Lecoutre and Breton, 2014), though these aspects are still largely unexplored.

The B3 vitamin and NAD^+^ precursor NR and the polyphenol resveratrol (RSV; 3,5,4′-trihydroxy-*trans*-stilbene) are two dietary compounds that enhance oxidative metabolism in adipose and other tissues when supplemented to adult rodents, possibly through their shared ability to activate sirtuin 1 (SIRT1) protein deacetylase (Lagouge et al., 2006; Canto et al., 2012; Alberdi et al., 2013; Andrade et al., 2014; Wang et al., 2015). Nutritional programming by RSV and NR has recently begun to be addressed (Zou et al., 2017; Ros et al., 2018; Serrano et al., 2018). We have shown that direct treatment of newborn mouse with mild doses of RSV or NR throughout lactation leads to sex-dependent long-term effects on the WAT thermogenic/oxidative phenotype. Signs of white-to-brown fat remodeling of iWAT in adulthood including smaller adipocyte size, enrichment in multilocular adipocytes, increased immunostaining against UCP1 and the respiratory chain protein component COXIV, and higher expression levels of genes related to brown fat determination, mitochondrial oxidative metabolism and beige markers (e.g., *Prdm16, Ppargc1b, Pparg, Cpt1b, Mfn2*, and *Slc27a1*) were found selectively in the treated male mice relative to sex-matched controls (Serrano et al., 2018). In parallel with these changes in WAT, RSV, and NR treated male mice displayed in adulthood better responses to an obesogenic high-fat diet than controls, such as a delayed body weight gain, a blunted increase in the plasma leptin-to-adiponectin ratio and a decreased lipolytic response (Serrano et al., 2018). However, the mechanisms behind the early programming of WAT energy metabolism related features by neonatal RSV and NR supplementations remain unknown.

Adipose tissue is a reservoir of immature progenitor cells which are part of the tissue stromovascular fraction (SVF). These cells undergo proliferation and differentiation (adipogenesis) *in vivo* to allow continuous renewal of the adipocytes in the fat depots throughout life, as well as hyperplasic adipose tissue expansion (i.e., increased adipocyte number) under conditions of positive energy balance, and are thus very important for adipose tissue homeostasis. Importantly, primary cultures established from the SVF of WAT can be used to unveil beige adipocytes (Petrovic et al., 2010), and these cultures reflect genetic differences in the capacity for brown-like adipogenesis in animals *in vivo* (Petrov et al., 2016). It is conceivable that nutritional influences during critical windows of WAT development condition the fate of adipogenesis from adipose tissue progenitor cells and, consequently, the metabolic features of adult WAT. The early postnatal period might be especially important for WAT programming. Subcutaneous (inguinal) WAT, in particular, basically forms in rodents coincident with the suckling period, after BAT (that develops mainly during the late fetal stage), and prior to visceral WAT (Cryer and Jones, 1979; Kozak, 2011).

In this work, we tested the hypothesis that mild supplementations with RSV or NR in early postnatal life can impact the programming of beige adipogenesis in progenitor cells resident in iWAT. To this end, gene expression related to thermogenesis and oxidative metabolism was studied in corresponding primary cultures established from the tissue SVF of young mice. The studies were conducted both in male and female mice, in view of previous reports of sexual dimorphism in thermogenic responses (Rodriguez and Palou, 2004; Valencak et al., 2017) and our previous characterization of RSV and NR treated offspring in adulthood, which pointed to sex-dependence of treatments effects (Serrano et al., 2018).

## Materials and Methods

### Animals and Experimental Design

Study protocols were approved by the Bioethical Committee of the University of the Balearic Islands (Ref. 3513, 26/03/2012). International standards for the use and care of laboratory animals were followed. Animals were housed at 22°C, with a 12 h light-dark cycle (lights on at 08:00), and free access to tap water and standard chow diet (type A03; 3.3 kcal/g, 8% calories from fat; Panlab, Barcelona, Spain). Virgin female NMRI mice (Charles River Laboratories, Barcelona, Spain) were mated. Each female was single-caged after mating. The morning in which newborn litters were found was designated as day 0. At day 2, litter size was adjusted to 12 pups per dam. Pups in four litters were randomly assigned to control or RSV group; in a separate experiment, pups in four additional litters were assigned to control or NR group (six pups per litter per group in both experiments). From postnatal day 2 (P2) to P20, the pups received daily orally 10–15 μL of either vehicle (water, control groups), a solution of RSV (Sigma, Madrid, Spain) or a solution of NR (Chemical Point, Diessenhofen, Germany), using a pipette. The amount of supplemented RSV was adjusted along the suckling period to meet a daily dose of 2 mg/kg body weight. RSV at 10–30 mg/kg animal/day decreases adiposity in adult rodents (Rivera et al., 2009; Alberdi et al., 2013) and we further applied a security factor considering the young age of the animals. The NR supplied ranged from 24 μg on P2 to 45 μg on P20, and was equivalent to ∼15-fold the total vitamin B3 ingested daily from maternal milk, considering the vitamin B3 content found in milk of in-home mouse dams [2378 ppb (Serrano et al., 2018)] and the daily milk intake of pups throughout lactation (Kojima et al., 1998). Animals were weaned and separated by sex on P21. The studies were performed separately in male and female mice. Body weight was periodically recorded from birth. Body composition was analyzed at P34, using an Echo MRI body composition analyzer (EchoMRI, LCC, Houston, TX, United States). Naso-anal length was also measured on P34, and used to calculate Lee’s obesity index (body weight in g^0.33^ × 1000/naso-anal length in cm). Energy intake from weaning until euthanization was estimated on a per-cage basis, from the actual amount of food consumed by the animals and its caloric equivalence. The animals were euthanized on P35, by decapitation, under fed conditions, within the first 2 h of the light cycle; half of the animals in each sex and treatment group (*n* = 5–6, from four different litters) were used to establish sex-separated adipose tissue primary cultures (see below), while the other half were used for tissue sampling for molecular analyses. Interscapular BAT and WAT depots (inguinal, epididymal, and retroperitoneal) were dissected in their entirety, weighed, snap-frozen in liquid nitrogen and stored at -80°C until processed. Glucose was measured from trunk blood using the Accu-Chek Aviva system (Roche Diagnostics). Adiposity index was calculated as the sum of all WAT depot mass divided per body weight and per cent.

### Adipocyte Primary Cultures

The SVF containing preadipocytes was obtained from iWAT depot and pooled (interscapular, cervical, and axillary) BAT depots of 35-day-old mice, following a previously described protocol (Petrovic et al., 2010). The cell pellet was suspended in culture medium (0.5 mL/animal for brown preadipocytes, 0.4 mL/animal for white preadipocytes) and seeded onto 6-well culture plates (2 mL culture medium and 0.2 mL of cell suspension per well). The culture medium was Dulbecco’s modified Eagle’s medium with 10% (v/v) newborn calf serum (Gibco, Thermo Fisher Scientific, Waltham, MA, United States), 4 nM insulin, 25 μg/mL sodium ascorbate, 10 mM HEPES, 4 mM glutamine, 50 units/mL penicillin, and 50 μg/mL streptomycin. From day 0 of culture, all wells were supplemented with 1 μM rosiglitazone (BioVision, Milpitas, CA, United States) to favor the recruitment of brown-like adipocytes (Petrovic et al., 2010). Medium was changed on day 1 and then every second day, except the day the cells were harvested. The cells were grown for 7 days at 37°C in an atmosphere of 8% CO_2_ in air. Adipogenic differentiation of the cells was regularly monitored through phase contrast microscopical examination. At harvesting, the percentage of cells showing intracellular lipid accumulation was ∼80% and 60% for the primary cultures established from, respectively, BAT and iWAT, as in previous reports from our group using the same primary culture protocol (Petrov et al., 2016) and without apparent differences between neonatal treatments (see representative microphotographs in Supplementary Figures S1, S2). Half of the cultures were exposed to 1 μM NA for the 2 h prior harvesting, to stimulate thermogenically competent cells and UCP1 expression (Petrovic et al., 2010).

### RNA Isolation, Retrotranscription, and Real-Time PCR

Total RNA was extracted using Tripure Reagent (Roche, Barcelona, Spain) according to the manufacturer’s instructions, followed by a sodium acetate/ethanol precipitation to purify nucleic acids. Isolated RNA was quantified using NanoDrop ND-1000 spectrophotometer (NanoDrop Technologies Inc., Wilmington, United States) and its integrity confirmed by agarose gel electrophoresis. Total RNA (0.25 μg/reaction) was reverse-transcribed using MuLV Reverse Transcriptase and random hexamers priming (Applied Biosystems, Madrid, Spain). Diluted cDNA template was used to perform PCR amplification of selected genes, along with specific forward and reverse primers (Sigma, Madrid, Spain), and Power SYBER Green PCR Master Mix (Applied Biosystems). Primers sequences are available on request. For Real-Time PCR the Applied Biosystems StepOnePlus Real-Time PCR Systems (Applied Biosystems) was used, with the following profile: 10 min at 95°C, followed by a total of 40 two-temperature cycles (15 s at 95°C and 1 min at 60°C). To verify the purity of the products, a melting curve was produced after each run. The threshold cycle was calculated by the instrument’s software (StepOne Software v2.2.2, Applied Biosystem) and the relative expression of each mRNA was calculated according to the 2^-ΔCt^ method, using 18S rRNA as reference gene.

### Statistical Analysis

Data are expressed as mean ± SEM. Statistical analyses were conducted separately by treatment (RSV and NR supplementation) and sex (male and female mice). Student’s *t*-test was used for single comparisons. Two-way ANOVA was used for analysis of treatments effects in primary adipocytes under basal and NA-stimulated conditions; *post hoc* comparisons were included when two-way ANOVA indicated an interactive effect between neonatal treatment and NA exposure of cultures. Threshold of significance was set at *p* < 0.05. IBM SPSS Statistics for Windows, version 19.0 (IBM Corp., Armonk, NY, United States) was used for the analyses.

## Results

### Neonatal RSV and NR Treatments Affected Beige Adipogenesis in iWAT Primary Cultures From Young Animals

To study the influence of treatments on the commitment of WAT preadipocytes toward the beige adipogenesis transcriptional program, expression levels of markers of brown/beige adipocyte determination and thermogenic function (*Ucp1, Cpt1b, Prdm16, Ppargc1a, Ppargc1b, Ppara*), and of purported beige adipocyte-selective transcriptional markers (*Tmem26, Hoxc9, Slc27a1*) were compared in differentiated iWAT primary cultures from RSV and NR mice and their respective controls. *Pparg* expression was also assessed, since the encoded product, PPARγ, is a master adipogenic factor that is also central to WAT browning (Ohno et al., 2012).

**FIGURE 1:**
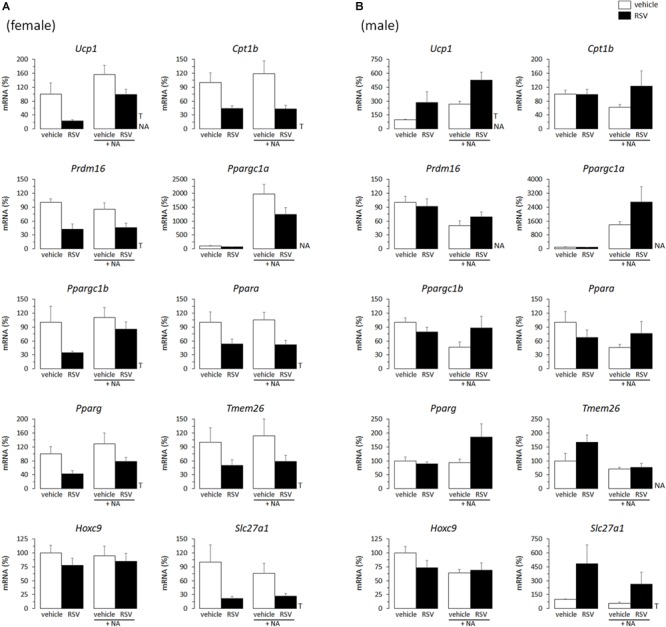
Gene expression (mRNA levels) of the indicated genes in primary adipocyte cultures established from inguinal white adipose tissue (iWAT) of female **(A)** and male **(B)** mice neonatally supplemented throughout the suckling period with resveratrol (RSV) and their corresponding controls given the vehicle (water). Cells were harvested on day 7 of culture, under basal conditions and after 2 h incubation with noradrenaline (NA). Data are the mean ± SEM of 5–6 animals/group; mean value of controls under basal conditions used as reference and set to 100%. Statistics (*p* < 0.05): T, treatment; NA, noradrenaline (two-way ANOVA).

Adipocytes differentiated from the iWAT SVF of RSV female mice showed a generalized decreased expression of transcripts of brown and beige adipocyte marker genes (namely: *Ucp1, Cpt1, Prdm16, Ppargc1b, Ppara, Tmem26, Slc27a1*), as well as of *Pparg*, when compared to primary adipocytes from control female mice (Figure 1A). On the contrary, primary adipocytes from RSV male mice showed increased expression levels of *Ucp1* and *Slc27a1* compared with corresponding controls (Figure 1B). *Ucp1* expression is the hallmark of the brown and beige adipocyte phenotype, and *Slc27a1* encodes a fatty acid transport protein (FATP1) that is highly expressed in BAT and other tissues actively oxidizing fatty acids and that is required for BAT thermogenesis (Wu et al., 2006). Furthermore, *Slc27a1* has been related to mitochondrial function in clonal white (3T3-L1) adipocytes (Wiczer and Bernlohr, 2009), and it is the only beige adipocyte transcriptional marker (out of 6 studied) found to be induced together with classical brown adipocyte marker genes in mouse WAT primary cultures following rosiglitazone exposure (Petrov et al., 2016). Additionally, trends to increased expression levels of most other genes related to oxidative metabolism/fatty acid oxidation assayed were apparent in the primary adipocytes derived from RSV male mice vs. controls under NA stimulated conditions (Figure 1B).

**FIGURE 2:**
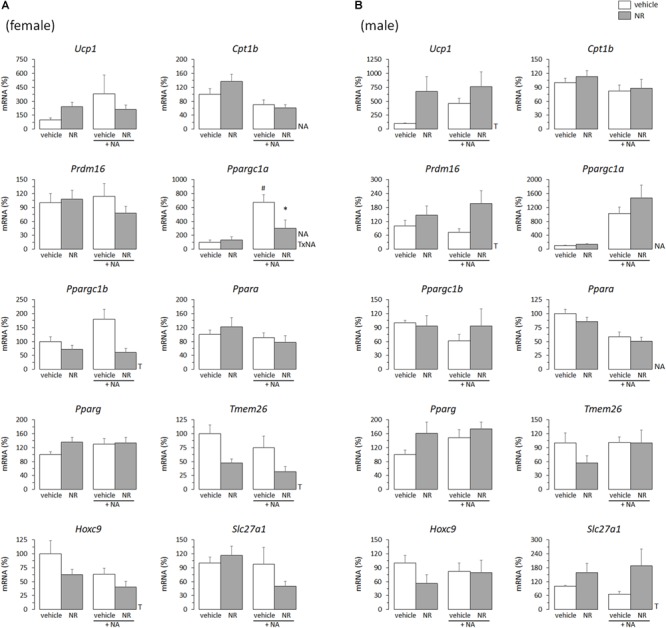
Gene expression (mRNA levels) of the indicated genes in primary adipocyte cultures established from inguinal white adipose tissue (iWAT) of female **(A)** and male **(B)** mice neonatally supplemented throughout the suckling period with nicotinamide riboside (NR) and their corresponding controls given the vehicle (water). Cells were harvested on day 7 of culture, under basal conditions and after 2 h incubation with noradrenaline (NA). Data are the mean ± SEM of 5–6 animals/group; mean value of controls under basal conditions used as reference and set to 100%. Statistics (*p* < 0.05): T, treatment; NA, noradrenaline; TxNA, interactive effect (two-way ANOVA); ^∗^NR vs. control, ^#^NA vs. basal (*t*-test).

The overall picture was similar for the NR treatment. *Ucp1* mRNA levels were increased in primary iWAT adipocytes derived from NR male mice but not female mice (Figure 2). Moreover, primary iWAT adipocytes from NR female mice showed a downregulated expression of several of the brown and beige transcriptional markers assessed (*Ppargc1a, Ppargc1b, Tmem26, Hoxc9*), whereas those from NR male mice showed an upregulated expression of *Slc27a1* – as for the RSV treatment – and also of *Prdm16* and *Pparg* (the latter with *p* = 0.073, two-way ANOVA) compared to corresponding controls (Figure 2). *Prdm16* encodes a transcriptional coactivator critical for WAT browning in postnatal life (Seale et al., 2011; Cohen et al., 2014), and agonism of PPARγ favors WAT browning *in vivo* and beige adipogenesis in primary adipose cultures (Fukui et al., 2000; Petrovic et al., 2010), possibly through stabilization of the PRDM16 protein (Ohno et al., 2012).

Half of the cultures were adrenergically stimulated prior harvesting to potentiate the emergence of thermogenically competent cells (Petrovic et al., 2010). Expected transcriptional responses to NA stimulation were found. In particular, *Ppargc1a* expression (encoding PGC1α) was induced by NA exposure in all iWAT primary cultures, regardless of sex of origin or neonatal treatment (Figure 1, 2). *Ucp1* expression was induced by NA exposure in the iWAT cultures from control and RSV mice regardless of sex, but not further induced by NA in the iWAT cultures from NR mice of either sex, which already overexpressed *Ucp1* compared to controls under the basal, non-stimulated, conditions (Figure 2). Apart from *Ppargc1a* and *Ucp1*, other brown/beige marker genes analyzed in iWAT primary cultures were not induced following NA exposure, but were rather unaffected or downregulated (Figure 1, 2). Down-regulation of genes in this class upon NA exposure was especially evident in the cultures derived from control male mice, and was attenuated in the cultures derived from RSV male mice (see results for *Cpt1b, Prdm16, Ppargc1b, Ppara, Hoxc9*, and *Slc27a1* expression in Figure 1B).

Altogether, these results indicated that the neonatal RSV and NR treatments had sex-dependent effects on the brown/beige transcriptional signature of iWAT primary cultures from young mice.

### Neonatal RSV and NR Treatments Affected Thermogenesis-Related Gene Expression in BAT Primary Cultures From Young Animals

**FIGURE 3:**
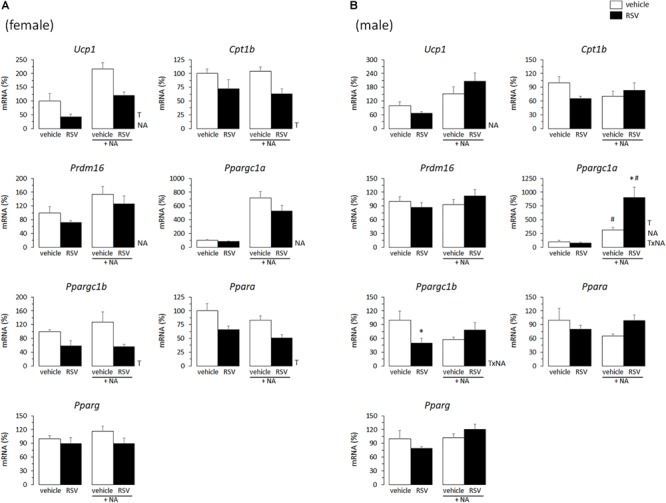
Gene expression (mRNA levels) of the indicated genes in primary adipocyte cultures established from brown adipose tissue (BAT) of female **(A)** and male **(B)** mice neonatally supplemented throughout the suckling period with resveratrol (RSV) and their corresponding controls given the vehicle (water). Cells were harvested on day 7 of culture, under basal conditions and after 2 h incubation with noradrenaline (NA). Data are the mean ± SEM of 5–6 animals/group; mean value of controls under basal conditions used as reference and set to 100%. Statistics (*p* < 0.05): T, treatment; NA, noradrenaline; TxNA, interactive effect (two-way ANOVA); ^∗^RSV vs. control, ^#^NA vs. basal (*t*-test).

**FIGURE 4:**
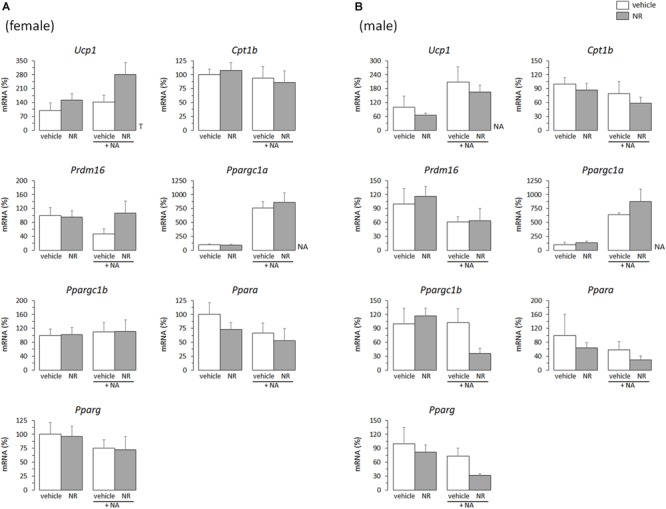
Gene expression (mRNA levels) of the indicated genes in primary adipocyte cultures established from brown adipose tissue (BAT) of female **(A)** and male **(B)** mice neonatally supplemented throughout the suckling period with nicotinamide riboside (NR) and their corresponding controls given the vehicle (water). Cells were harvested on day 7 of culture, under basal conditions and after 2 h incubation with noradrenaline (NA). Data are the mean ± SEM of 5–6 animals/group; mean value of controls under basal conditions used as reference and set to 100%. Statistics (*p* < 0.05): T, treatment; NA, noradrenaline (two-way ANOVA).

In adult animals, it is common that the same stimuli that induce WAT browning also induce BAT recruitment (Bonet et al., 2013, 2017). Thus, effects of the *in vivo* neonatal treatments on thermogenic gene expression in primary BAT adipocytes from young mice were studied, to check for potential parallelisms between results in iWAT- and BAT-derived cultures. Compared with levels in cultures from sex-matched controls, the mRNA levels of *Ucp1, Cpt1b, Ppargc1b*, and *Ppara* were downregulated in the BAT primary cultures from RSV female mice, but not (except for *Ppargc1b* expression under basal conditions) in those from RSV male mice (Figure 3A,B). On the contrary, BAT cultures from RSV male mice more readily induced *Ucp1* and *Ppargc1a* mRNA levels following NA stimulation, and showed increased *Ppargc1a* and *Ppara* mRNA levels under NA-stimulated conditions compared with cultures from control male mice. Furthermore, upon NA stimulation, *Ppargc1b* expression levels decreased in the BAT cultures from control male mice but not in those from RSV male mice. Thus, the overall effect of neonatal *in vivo* RSV treatment on BAT primary adipocyte cultures from young mice resembled that in iWAT primary adipocyte cultures, with thermogenic gene expression being potentiated in the cultures derived from male mice and attenuated in the cultures derived from female mice. Somewhat oppositely, neonatal NR treatment associated with higher *Ucp1* expression selectively in the female mice-derived BAT cultures, whereas expression of other genes assayed (*Cpt1b, Prdm16, Ppargc1a, Ppargc1b, Ppara, Pparg*) was not significantly affected by neonatal NR treatment in either sex (Figure 4A,B). Expected responses to NA, namely induction of *Ucp1* and *Ppargc1a* expression, were present in all BAT primary cultures regardless of sex of origin or treatment (Figure 3, 4).

### Effects of Neonatal RSV and NR on Early Postnatal Growth

Eventual effects of treatments on early postnatal growth were evaluated by monitoring body weight evolution from birth; body length (nasal-to-anal distance), Lee index and body composition (by EchoMRI) at P34; and tissue/organ weights at euthanization at P35. RSV treatment had no significant effect on any of these parameters, neither in male nor in female animals (Table 1). As for the NR treatment, treated male mice, but not females, were slightly but significantly heavier (by ∼6%) and had higher (by ∼20%) body fat content and fat/lean ratio at P34 compared to sex-matched controls (Table 2). Consistent with these body composition results, NR male mice, but not females, displayed after dissection a trend to higher gonadal WAT mass (*p* = 0.090) (Table 2). Cumulative energy intake from weaning until P30 was significantly lower (by ∼4%) in the NR animals of both sexes relative to sex-matched controls (Table 2), and showed a trend to be lower (also by ∼4%, *p* = 0.076) in the RSV male mice, but not the RSV female mice. Fed blood glucose levels at P35 were unaffected by treatments in either sex (not shown). Collectively, these results indicate that the neonatal RSV and NR supplementations applied did not compromise the animals’ growth during early postnatal life, and had only a minor impact on it.

**Table 1:** Biometric parameters in mice receiving resveratrol (RSV) supplementation throughout lactation and their corresponding control littermates.

	Female		Male	
	Vehicle	RSV	Vehicle	RSV
**Body weight evolution (g)**				
Day 3	2.7 ± 0.09	2.7 ± 0.17	2.7 ± 0.07	2.7 ± 0.07
Day 12	7.5 ± 0.19	7.2 ± 0.18	7.5 ± 0.16	7.2 ± 0.16
Day 21	13.8 ± 0.41	13.8 ± 0.41	14.9 ± 0.43	14.7 ± 0.38
Day 30	23.7 ± 0.44	23.6 ± 0.65	27.9 ± 0.83	27.6 ± 0.67
Cumulative energy intake from day 21 to day 30 (kcal/mice)	115 ± 1.8	117 ± 0.3	143 ± 1.2	137 ± 2.2
**Body composition (day 34)**				
Body weight (g)	26.6 ± 0.55	26.3 ± 0.47	31.4 ± 0.73	30.3 ± 0.53
Fat mass (g)	4.0 ± 0.31	4.0 ± 0.23	3.8 ± 0.26	3.4 ± 0.17
Lean mass (g)	20.0 ± 0.39	19.7 ± 0.35	25.2 ± 0.59	24.5 ± 0.40
Fat mass (g/100 g bw)	14.9 ± 0.97	15.3 ± 0.81	11.9 ± 0.60	11.2 ± 0.43
Lean mass (g/100 g bw)	75.1 ± 0.79	74.9 ± 0.76	80.6 ± 1.36	81.0 ± 0.36
Ratio fat/lean mass	0.168 ± 0.013	0.172 ± 0.010	0.128 ± 0.007	0.120 ± 0.005
Naso-anal length (cm)	9.2 ± 0.09	9.2 ± 0.07	9.5 ± 0.11	9.6 ± 0.13
Lee index (g^0.33^1000/cm)	326 ± 03.1	323 ± 2.2	332 ± 2.2	324 ± 3.7
**Final animal/tissue weights (day 35)**				
Body weigth (g)	26.2 ± 0.47	27.1 ± 0.65	31.4 ± 0.98	31.4 ± 0.63
Inguinal WAT (g/100g bw)	1.39 ± 0.10	1.58 ± 0.15	1.11 ± 0.07	1.15 ± 0.08
Gonadal WAT (g/100g bw)	1.14 ± 0.19	1.42 ± 0.23	1.23 ± 0.15	1.32 ± 0.10
Interscapular BAT (g/100g bw)	0.60 ± 0.04	0.72 ± 0.04	0.53 ± 0.04	0.60 ± 0.05
Liver (g/100g bw)	2.79 ± 1.11	2.09 ± 1.07	3.11 ± 1.26	3.30 ± 1.34
Gastrocnemius SM (g/100 g bw)	0.80 ± 0.04	0.90 ± 0.03	0.89 ± 0.04	0.88 ± 0.04

*Data on body weight evolution, energy intake, and body composition correspond to the mean ± SEM of 11–12 animals per sex and treatment (vehicle or RSV); data on final animal/tissue weights are the mean ± SEM of 5–6 animals per sex and treatment. bw, body weight; SM, skeletal muscle. No significant differences in any of the Table’s parameters were revealed by Student’s *t*-test.*

**Table 2:** Biometric parameters in mice receiving nicotinamide riboside (NR) supplementation throughout lactation and their corresponding control littermates.

	Female		Male	
	Vehicle	NR	Vehicle	NR
**Body weight evolution (g)**				
Day 3	2.9 ± 0.08	2.8 ± 0.07	3.1 ± 0.07	3.1 ± 0.06
Day 12	7.1 ± 0.22	6.9 ± 0.21	7.0 ± 0.26	7.4 ± 0.21
Day 21	12.4 ± 0.37	12.0 ± 0.32	12.5 ± 0.50	13.2 ± 0.39
Day 30	19.7 ± 1.37	19.4 ± 1.21	21.3 ± 1.31	23.5 ± 1.62
Cumulative energy intake from day 21 to day 30 (kcal/mice)	128 ± 0.8	122 ± 1.6^∗^	159 ± 1.6	153 ± 1.6^∗^
**Body composition (day 34)**				
Body weigth (g)	25.6 ± 0.38	26.1 ± 0.39	30.3 ± 0.48	32.0 ± 0.58^∗^
Fat mass (g)	2.9 ± 0.16	2.7 ± 0.16	2.4 ± 0.16	3.0 ± 0.22^∗^
Lean mass (g)	19.3 ± 0.34	19.7 ± 0.24	24.8 ± 0.39	25.8 ± 0.47
Fat mass (g/100 g bw)	11.4 ± 0.58	10.5 ± 0.49	7.8 ± 0.45	9.4 ± 0.60^∗^
Lean mass (g/100 g bw)	75.2 ± 0.55	75.3 ± 0.40	81.7 ± 0.49	80.7 ± 0.68
Ratio fat/lean mass	0.152 ± 0.009	0.139 ± 0.007	0.096 ± 0.006	0.118 ± 0.009^∗^
Naso-anal length (cm)	9.0 ± 0.10	8.9 ± 0.11	9.3 ± 0.07	9.3 ± 0.08
Lee index (g^0.33^1000/cm)	328 ± 4.1	336 ± 4.4	336 ± 2.8	339 ± 3.2
**Final animal/tissue weights (day 35)**				
Body weigth (g)	27.6 ± 0.82	27.4 ± 0.83	33.2 ± 0.60	35.0 ± 0.79
Inguinal WAT (g/100g bw)	1.41 ± 0.15	1.38 ± 0.15	1.08 ± 0.08	1.19 ± 0.04
Gonadal WAT (g/100g bw)	1.12 ± 0.24	0.93 ± 0.16	1.12 ± 0.14	1.47 ± 0.12
Interscapular BAT (g/100g bw)	0.60 ± 0.04	0.66 ± 0.07	0.63 ± 0.04	0.62 ± 0.03
Liver (g/100g bw)	2.18 ± 1.13	2.89 ± 1.17	3.44 ± 1.16	2.68 ± 1.44
Gastrocnemius SM (g/100 g bw)	0.88 ± 0.03	0.89 ± 0.01	0.94 ± 0.02	0.96 ± 0.03

*Data on body weight evolution, energy intake and body composition correspond to the mean ± SEM of 11–12 animals per sex and treatment (vehicle or RSV); data on final animal/tissue weights are the mean ± SEM of 5–6 animals per sex and treatment. bw, body weight; SM, skeletal muscle. ^∗^*p* < 0.05 vs. vehicle, Student’s *t*-test.*

### Effects of Neonatal RSV and NR Treatments on Targeted Gene Expression in iWAT of Young Mice

**FIGURE 5:**
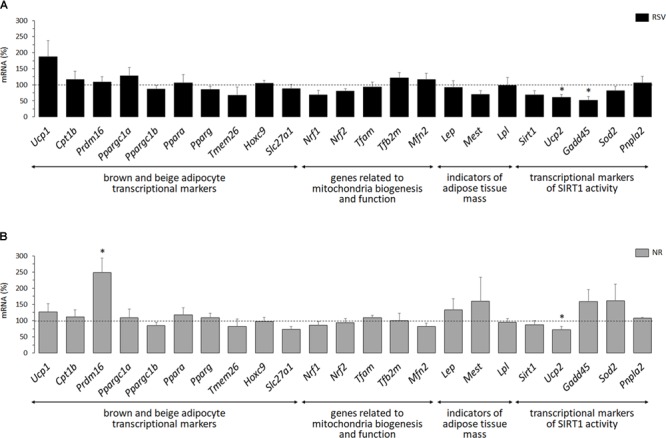
Gene expression (mRNA levels) of the indicated genes in inguinal white adipose tissue (iWAT) of 35-day-old male mice neonatally supplemented throughout the suckling period with resveratrol (RSV, **A**) or nicotinamide riboside (NR, **B**) relative to corresponding controls given the vehicle (water; dotted line). Data are the mean ± SEM of 5–6 animals/group. Statistics (*p* < 0.05): ^∗^RSV or NR vs. control (*t*-test).

The same panel of brown and beige adipocyte transcriptional markers analyzed in the iWAT primary cultures was analyzed in the iWAT depot of 35-day-old RSV and NR male mice and their respective control littermates (Figure 5). Genes related to mitochondria biogenesis and function were additionally analyzed in the iWAT tissue, since expression of genes in this category (*Nrf1, Nrf2, Tfam, Tfb2m, Mfn2*) is increased at adult age in iWAT of RSV and NR neonatally treated male mice compared to control littermates (Serrano et al., 2018). Of the genes analyzed, the only significant difference from expression levels in controls was a greater than 2-fold increase in *Prdm16* mRNA levels in the iWAT of young NR male mice (Figure 5). *Lep, Mest*, and *Lpl* mRNA levels in iWAT, which were used as indicators of adipose tissue mass and expandability, were unaffected by treatments (Figure 5), in good concordance with the lack of effect of treatments on iWAT depot mass (Tables 1, 2). mRNA expression levels of a selection of genes in the above categories (*Ucp1, Slc27a1, Mfn2, Lpl*) were analyzed in iWAT of young (P35) female mice and found to be unaffected by treatments (data not shown).

Finally, because RSV and NR can both enhance energy metabolism through the activation of SIRT1 (Canto et al., 2012; Park et al., 2012; Price et al., 2012), transcriptional markers of SIRT1 activity were assessed in iWAT of young animals. SIRT1 represses *Ucp2* gene transcription (Bordone et al., 2006) and deacetylates FOXO1 transcription factor, resulting in higher expression levels in cells of FOXO1 target genes such as *Gadd45, Sod2*, and *Atgl* (Calnan and Brunet, 2008; Chakrabarti et al., 2011). Consistent with neonatal RSV and NR treatments activating SIRT1 specifically in iWAT of male mice, *Ucp2* mRNA levels were significantly decreased in iWAT of both groups of treated male mice relative to controls (Figure 5), but not in the iWAT depot of treated female mice (controls, 100 ± 14 and 100 ± 21; treated 134 ± 63 and 92 ± 21, for the RSV and NR treatments, respectively). However, none of the three FOXO1 target genes analyzed was significantly induced by the experimental treatments in iWAT of young male mice, and expression of *Gadd45* was even significantly downregulated in the RSV male mice. *Sirt1* expression was also measured, and a trend to down-regulation of its mRNA levels was apparent in the RSV male mice (*p* = 0.053). Overall, from the transcriptional analysis performed, we conclude that there is little evidence of iWAT browning or SIRT1 activation in iWAT of young male mice receiving neonatal RSV or NR supplementation.

## Discussion

Modulation of the browning potential of adipose tissues through the intake of food bioactives in early life is a potential, yet still largely unexplored, strategy to enhance metabolic health in later life (Palou et al., 2013). Adipose progenitor cells resident in fat depots are likely to be natural depositors of programming information from dietary and other cues in critical life stages, making the primary culture an attractive model for studies in the field of nutritional programming of adipose tissue cellular and metabolic features. Using this model, we show here that the intake of mild supraphysiological doses of RSV or NR during the suckling period impacts adipocyte precursor cells resident in subcutaneous WAT of young mice and affects the commitment of these cells to the beige adipogenesis transcriptional program in a sex-dependent manner, down-regulating it in the female mice and up-regulating it in the male mice. As expected considering the rather mild dietary treatments applied, gene expression changes observed were relatively small, yet they affected different functionally related genes concertedly. Interestingly, long-term consequences of neonatal RSV and NR treatments under the same conditions as those used here include signs of iWAT-to-BAT remodeling and better responses to an obesogenic high-fat diet in adulthood selectively in the male mice (Serrano et al., 2018). A causal link between the sex-dependent effects on iWAT preadipocyte differentiation revealed here in the primary cultures and the sex-dependent long-term effects on iWAT features and benefits against metabolic challenge found *in vivo* in adulthood is thus suggested.

Our studies are first to examine nutritional programming of adipose tissue metabolic features by NR and RSV supplements given directly to the lactating pups. Programming effects of supplemental NR has not previously been addressed, to our knowledge. In the case of RSV, maternal supplementation with this compound was previously shown to enhance brown/beige adipocyte function in the male offspring of high-fat diet-fed dams, as indicated by WAT and BAT analysis and systemic measurements (Zou et al., 2017). However, in this previous study RSV was included in the dam’s diet at a rather high dose (0.2% in the diet, ∼200 mg/kg body weight per day) and throughout pregnancy and lactation, whereas we here used a mild, precise dose of RSV (2 mg/kg body weight per day) supplied directly to the pups. Additionally, only effects on the male offspring of RSV-supplemented dams were previously reported (Zou et al., 2017), whereas our study included both male and female offspring, thus allowing to unveil sex-dependent effects.

Differences in WAT browning capacity due to early metabolic imprinting might be better revealed in young animals through the establishment of WAT primary cultures, which can be readily stimulated with rosiglitazone and NA in order to recruit and activate beige adipocytes in them, than through direct WAT depot analysis (especially in the absence of extra *in vivo* challenges, such as cold-exposure). In fact, whereas brown/beige gene expression was enhanced in the derived primary cultures, iWAT of young (P35) RSV and NR treated male mice did not show an obvious brown fat-like gene expression signature: out of the fifteen brown/beige markers and mitochondria-related genes assayed, only *Prdm16* in the NR-treated male group was found to be upregulated at P35 compared to levels in controls. However, up-regulation of many of these genes in the iWAT of mice neonatally treated with RSV or NR is apparent at adult age (P164), after regular diet feeding and/or high-fat diet feeding (Serrano et al., 2018). Taken together, we interpret these results as indicating that, with time–after rounds of preadipocyte proliferation and *de novo* adipogenesis *in vivo*, linked to aging and eventual obesogenic diet feeding–the greater commitment to brown-like adipogenesis translates into a greater appearance of BAT-like properties in the iWAT of treated male mice. In contrast, the decreased capacity for beige adipogenesis found here in iWAT primary cultures from young RSV and NR treated female mice does not translate into a generalized lower thermogenic/oxidative gene expression in the adult female iWAT, in which most of these markers are unaffected (Serrano et al., 2018). Challenging the adult animals with a strong acute pro-browning stimulus, such as cold-exposure, may be required to reveal inhibitory programming of WAT browning *in vivo*.

Whereas for iWAT of male animals there is a good concordance between observed effects of neonatal RSV and NR treatments on primary adipose cultures established at young age and observed adipose depot features at adult age, this is not the case for BAT. For instance, neonatal RSV supplementation enhanced the expression of brown marker genes in the BAT primary cultures (this work) but not in BAT of male mice in adulthood, where if anything there was decreased expression (Serrano et al., 2018). The difference may relate to the fact that our treatment period is coincident with iWAT rather than BAT development during ontogeny (Kozak, 2011). Thus, we suggest that observed effects of neonatal RSV and NR treatments on BAT preadipocyte adipogenesis in primary culture are likely a transient reflect of recent treatment, whereas effects on iWAT preadipocyte adipogenesis are likely to be persistent and of programming nature, when the treatment is performed in the specific developmental time window of lactation.

Previous studies on the nutritional programming of adipose tissue features have mainly focused on the influence of maternal diet during pregnancy and lactation, studying factors such as maternal total energy intake (Garcia et al., 2011; Palou et al., 2015), diet macronutrient composition (Dumortier et al., 2017) or dietary fat quality (Priego et al., 2013; Kodde et al., 2017). Specific nutrients/bioactives such as RSV itself (Zou et al., 2017; Ros et al., 2018), grape seed procyanidins (del Bas et al., 2015), or leptin (Konieczna et al., 2015; Szostaczuk et al., 2017), among others, have been tested in this context mainly for their ability to counteract detrimental effects of maternal malnutrition, as in calorie restricted or high-fat diet fed dams. Early postnatal nutritional programming by specific nutrients/bioactives independently of maternal diet has been much less studied, despite its biological interest and its translational interest for the baby food market. There are studies of this kind for vitamin A (Granados et al., 2013; Musinovic et al., 2014), n-3 long-chain polyunsaturated fatty acids (Oosting et al., 2010), and leptin (Priego et al., 2010), for instance, but those studies generally focused on effects on WAT expansion, and did not specifically address effects on the WAT browning potential. Our findings are among first proof-of-concept that the fate of preadipocytes in WAT toward the beige adipogenesis transcriptional program can be influenced by the intake of specific nutrients and food bioactives in the early postnatal life, independently of maternal diet. Another novel aspect of the present work is the use of the primary culture model, which so far has been little exploited in the field of nutritional programming, despite its potential relevance for this field. To our knowledge, only one recent report has used a similar approach, to demonstrate that neonatal overfeeding affects the differentiation capacities of adipose tissue mesenchymal stem cells (Dias et al., 2018).

Sexual dimorphism regarding the BAT thermogenic system is well-known (reviewed in Rodriguez and Palou, 2004; Valencak et al., 2017). Female rats possess more BAT of higher thermogenic capacity compared to male rats, and the same appears to be the case in humans (Valencak et al., 2017). BAT thermogenesis is more easily activated by cold in female rats (Rodriguez and Palou, 2004) and in women (van den Beukel et al., 2015) than in male counterparts, but less efficiently activated by excess palatable food intake in female than in male rats (Rodriguez and Palou, 2004). Sex differences in the capabilities for browning of WAT have also been described, with greater levels of WAT browning in female than male mice (Kim et al., 2016) and in women than in men (Barquissau et al., 2018). Our results extend sexual dimorphism of the WAT browning phenomenon to its developmental programming in response to dietary factors. Additionally, from an operational point of view, results herein underscore that sex-dependent responses can be unveiled in primary cultures, a fact often neglected in studies using this model system.

Programming effects of RSV and NR neonatal treatments observed in this work may result from epigenetic changes in adipocyte precursor cells subsequent to treatment effects at the iWAT level and/or the central level, on neuronal circuitries impinging on the developing adipose tissue. Such actions may involve SIRT1 activation, since (i) RSV and NR can both activate SIRT1, albeit through distinct mechanisms (Canto et al., 2012; Park et al., 2012; Price et al., 2012), (ii) the activity of SIRT1 favors BAT thermogenesis and WAT browning [(Boutant and Canto, 2016) and references therein], and (iii) SIRT1 interacts with epigenetic mechanisms including DNA methylation (Ions et al., 2013). However, besides SIRT1, both RSV and NR have distinct, non-overlapping biological targets for interaction, and it is to be noted that observed effects of neonatal NR and RSV treatments, though similar, were not identical, neither in the short-term (this work) nor in the long-term (Serrano et al., 2018). Moreover, we found little evidence of activation of SIRT1 in iWAT of young treated mice, where of four transcriptional markers of SIRT1 activity assayed, only changes in *Ucp2* expression in the expected sense (Bordone et al., 2006) relative to controls were present. Further studies on the mechanisms behind programming by neonatal RSV and NR treatments of energy metabolism in adipose tissues and other key tissues in whole body energy homeostasis are warranted.

## Author Contributions

MLB, JR, AMR, and AP conceived and designed the work. MA-K managed the animals. MA-K and AR established the primary cultures. MA-K and AS acquired the data. MLB and JR wrote the article. All authors analyzed and interpreted the data, revised the manuscript critically, and gave final approval of the version to be published.

## Conflict of Interest Statement

The authors declare that the research was conducted in the absence of any commercial or financial relationships that could be construed as a potential conflict of interest.

## Abbreviations

BAT: brown adipose tissue
iWAT: inguinal WAT
NA: noradrenaline
NR: nicotinamide riboside
RSV: resveratrol
WAT: white adipose tissue
