# Supplementary material for: Neonatal Resveratrol and Nicotinamide Riboside Supplementations Sex-Dependently Affect Beige Transcriptional Programming of Preadipocytes in Mouse Adipose Tissue
Source: Front Physiol. 2019 Feb 8;10:83. doi: 10.3389/fphys.2019.00083 (PMC6375887; doi:10.3389/fphys.2019.00083)

Supplementary Fig. 1 - Representative microphotographs at the day of harvesting (day 7) of interscapular BAT and inguinal WAT primary cell cultures established from young female and male mice treated with vehicle or resveratrol (RSV) during lactation

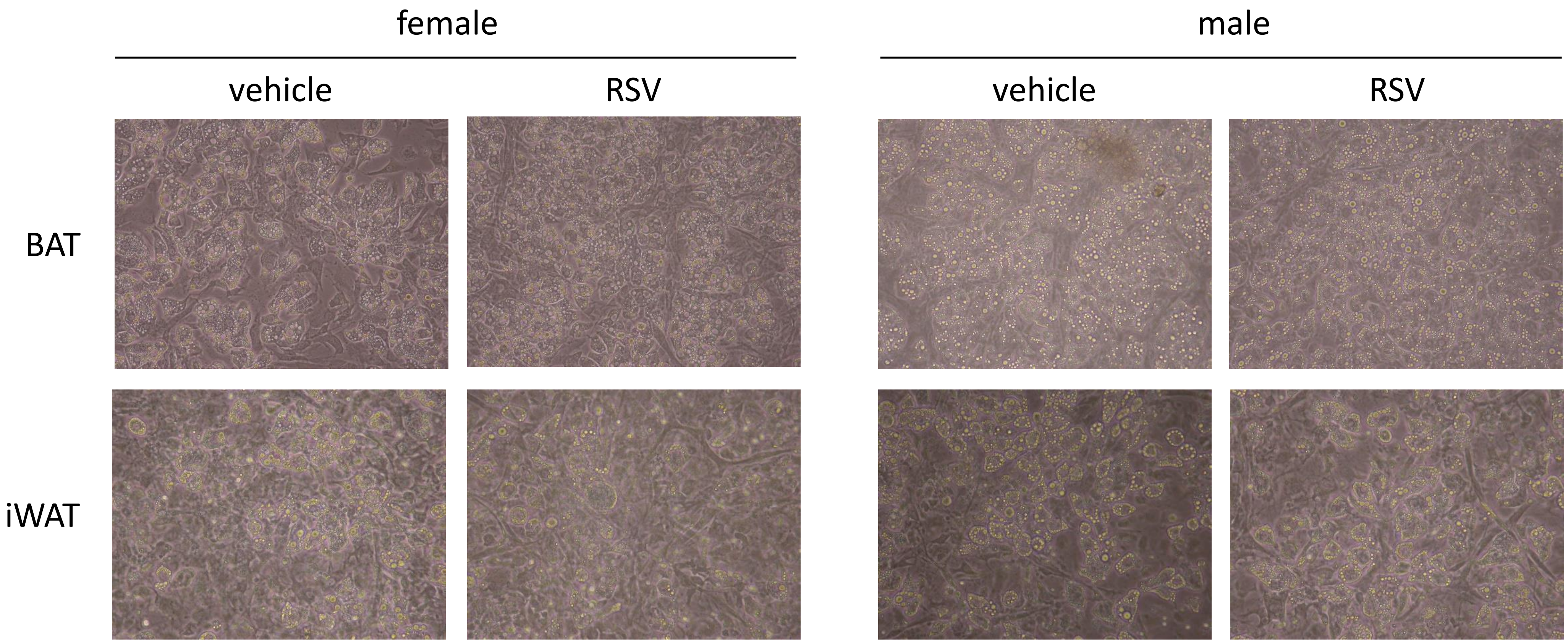

Supplementary Fig. 2 - Representative microphotographs at the day of harvesting (day 7) of interscapular BAT and inguinal WAT primary cell cultures established from young female and male mice treated with vehicle or nicotinamide riboside (NR) during lactation

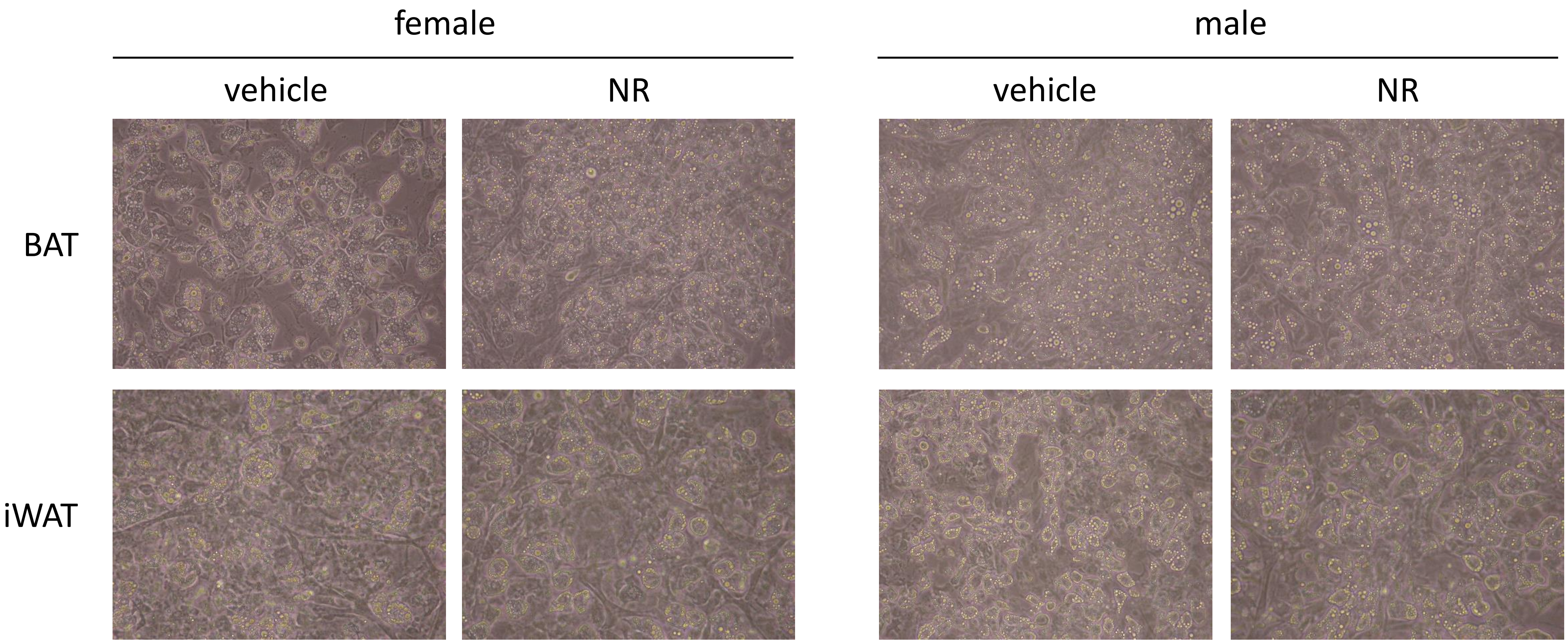

Supplement: Supplementary file 1 [file Image_1.pdf]
